# The resonance® metallic ureteral stent in the treatment of malignant ureteral obstruction: a prospective observational study

**DOI:** 10.1186/s12894-019-0569-y

**Published:** 2019-12-27

**Authors:** Jun Miyazaki, Mizuki Onozawa, Satoshi Takahashi, Yuka Maekawa, Mitsuru Yasuda, Koichiro Wada, Yuji Maeda, Takuro Masaki, Akito Yamaguchi, Masahiko Suzuki, Yasuyuki Sakai, Tomokazu Kimura, Manabu Takai, Kensaku Seike, Takahiko Hashimoto, Shingo Yamamoto

**Affiliations:** 1 Department of Urology, International University of Health and Welfare, 6-1-14 Kounodai, Ichikawa City, Chiba 272-0827 Japan; 2 Department of Infection Control and Laboratory Medicine, Sapporo Medical University School of Medicine, S1 W16, Chuo-ku, Sapporo, 060-8543 Japan; 3 Department of Urology, Gifu University Graduate School of Medicine, 1-1 Yanagido, Gifu City, 501-1194 Japan; 4 Department of Urology, Okayama University Graduate School of Medicine, Dentistry and Pharmaceutical Sciences, 2-5-1 Shikata-cho, Kita-ku, Okayama, 700-8558 Japan; 5 Department of Urology, Public Central Hospital of Matto Ishikawa, 3-8 Kuramitsu, Hakusan-shi, Ishikawa 924-0865 Japan; 6 Department of Urology, Harasanshin Hospital, 1-8 Taihaku-machi, Hakata-ku, Fukuoka City, Fukuoka, 812-0033 Japan; 7 Department of Urology, Kasumigaura Medical Center, 2-7-14 Shimotakatsu, Tsuchiura City, Ibaraki 300-0812 Japan; 8 Department of Urology, National Cancer Center East, 6-5-1 Kashiwanoha, Kashiwa-shi, Chiba 277-8577 Japan; 9 Department of Urology, Faculty of Medicine, University of Tsukuba, Tennodai 1-1-1, Tsukuba City, Ibaraki 305-8575 Japan; 10 Department of Urology, Kizawa Memorial Hospital, 590 Shimokobi, Kobicho, Minokamo, Gifu, 505-8503 Japan; 11 Department of Urology, Chuno Kosei Hospital, 5-1 Wakakusadori, Seki-city, Gifu, 501-3802 Japan; 12 Department of Urology, Hyogo College of Medicine, 1-1 Mukogawa-cho, Nishinomiya City, Hyogo 663-8501 Japan

**Keywords:** Metal stent, Malignant ureteral obstruction, Ureteral stent, Metallic ureteric stenting, Resonance stent

## Abstract

**Background:**

To study the outcomes and experiences of using metallic stents in treating patients with malignant ureteral obstruction (MUO), we examined the effects of metallic ureteral stenting using the Cook Resonance® stent in the treatment of MUO.

**Methods:**

All patients who had a Resonance metallic stent inserted between April 2015 and March 2018 at one of multiple facilities were prospectively observed with a 1-year follow-up. The primary outcome was the patency rate of the metallic ureteral stent. The secondary outcomes included the complications (e.g., infection and fever).

**Results:**

Although stent insertion was attempted in 50 patients, the stent could not be inserted as a ureteral stent in three patients due to severe ureteral stricture, and one ureteral cancer patient was excluded from the analysis. The remaining 46 patients’ median age was 67 years (range 28–85 years) (16 males, 30 females). Twenty-four patients died during the study; their median survival time was 226 days. The median follow-up period for the censored patients was 355 days (range 16–372 days), and just seven patients were still alive without Resonance failure > 1 year later. The women’s IPSS scores tended to be lower than those of the men. Regarding the OABSS score, although the women’s total score tended to be low, the difference between the men’s and women’s scores was nonsignificant. The bacteria detected from urine culture after stent insertion were more gram-positive than gram-negative.

**Conclusion:**

Metallic ureteric stenting using the Resonance stent is safe and effective for treating MUO. Subjective symptoms were relatively less in the female patients.

## Background

A malignant ureteral obstruction (MUO) can be caused by a malignancy that compresses the ureter externally. MUO has been reported to be an indicator of poor prognosis, and the median life expectancy of patients with metastatic cancer that causes a ureteral obstruction is generally < 1 year [1–3]. Immediate urinary diversion using a ureteral stent and the preservation of renal function are required for an MUO, especially when further chemotherapy is considered [4, 5].

The Resonance® Metallic Ureteral Stent (hereafter, ‘the Resonance;’ Cook Medical, Bloomington, IN, USA) is the first metal ureteral stent (MRI-compatible) available for use in Japan. Made of a Ni-Co-Cr-M alloy (corrosion-resistant and resistant to calculus adhesion), the Resonance has a tightly wound wire coil structure, with no side hole openings at either end. The Resonance enables continuous drainage as urine passes through the stent from gaps in the tightly wound coil and exits the stent; this stent is extremely resistant to collapsing and kinking from extrinsic pressure [6]. Since Borin et al. reported the initial experiences with the Resonance metallic stent [7], it was approved in 2007 in many countries. It is resistant to migration and dislodgement because it is placed along the entire length of the ureter. In addition, like the existing products, the Resonance is easy to deploy and remove, and problems with long-term placement (e.g., stent occlusion, calculus formation on the surface, migration, and dislodgement) have been rectified, enabling the urinary tract to be secured for a maximum of 12 months without changing the stent, even in patients with an MUO.

The Resonance has an occluded end design, which results in urine draining through small gaps between the spirally wound coils [5]. This provides reduced stent encrustation that requires only an annual exchange. The Resonance was also confirmed to be more cost-effective than standard polymer stenting [8]. However, the published experience with the Resonance is limited and includes case series with small numbers of patients [9–17]. We conducted the present study to evaluate the efficacy and safety of the Resonance in the treatment of MUO, in a multicenter prospective study. We report the treatment outcomes based on our multicenter prospective study cohort of 50 patients with indwelling metallic stents against MUO, and we identify an indicator of stent patency in these patients.

## Patients and methods

This study protocol was approved by the Tsukuba University Protocol Review Committee (H26–168), the Tsukuba Clinical Research & Development Organization (T-CReDO), and the institutional review board of each participating hospital before the initiation of the study. This study was performed in accordance with the international ethical recommendations stated in the Declaration of Helsinki [18] and the Japanese Ethical Guidelines for Epidemiological Research. The T-CReDO conducted central monitoring to ensure the integrity of the data submission and patient eligibility and on-schedule study progress.

### Study design

This prospective observational study was conducted at 10 centers by volunteer members of or related to the Stent Committee of the Japanese Society of Endourology. The clinical data accumulation was performed by T-CReDO. Cook Medical Co. paid T-CReDO for the data collection costs.

The inclusion criterion for the patients with a first ureteral obstruction due to extrinsic underlying disease was a ureteral obstruction due to a gastrointestinal, gynecological, or urological tumor (excluding cases in which the primary disease was ureteral cancer or renal pelvic cancer). Between December 2014 and March 2018, we enrolled 50 patients with MUOs who were referred to a participating hospital for retrograde stenting. The study endpoint was the patency rate of the metallic ureteral stent. The secondary endpoints were the complications, e.g., infection and fever. When a patient’s renal function was shown to be impaired by a blood test or imaging test, the patient’s case was classified as stent obstruction. Stent failure was also defined as unanticipated stent exchange or nephrostomy placement for signs of ureteral obstruction based on imaging (ultrasound of CT scans) conducted every 3 months or on a blood test (creatinine).

All Resonance stents were placed in standard retrograde fashion with the use of X-ray guidance, a guidewire, and a coaxial inner sheath and outer catheter under spinal anesthesia or local anesthesia. The stent was inserted through the sheath after the guidewire and inner catheter were removed. The length of the Resonance was determined depending on each institution’s guideline or by the treating physician. After the placement of the Resonance stent, the patients were followed for up to 1 year or until obstruction of the ipsilateral ureter, death, or an adverse event requiring management occurred.

On the day before the stent placement and at 3, 6, 9, and 12 months post-placement, follow-up imaging, creatinine measurement, urine cultures, and testing for the patient’s International Prostate Symptom Score (IPSS) and Overactive Bladder Symptom Score (OABSS) were carried out in stable patients. For the actual follow-up visit date, deviations of approx. 4 weeks from the scheduled date were allowed. The IPSS, developed by Barry et al. [19], was initially used to assess the symptom severity of benign prostatic hyperplasia [20]. It was subsequently noted that the IPSS is neither sex-specific nor disease-specific for benign prostatic hyperplasia [21, 22]. Many clinician have already used the IPSS in daily practice and/or epidemiologic surveys of lower urinary tract symptoms (LUTS) in female patients [23, 24].

The IPSS can be subdivided into the IPSS voiding subscore (IPSS-V) and the IPSS storage subscore (IPSS-S) [25, 26]. The IPSS-V is the sum of the answers to question 1 (incomplete emptying), question 3 (intermittency), question 5 (weak stream), and question 6 (straining to void). The IPSS-S is the sum of the answers to question 2 (frequency), question 4 (urgency), and question 7 (nocturia) (the sum of the voiding and post-void symptom subscores is obtained). When patients were alive > 1 year after stent placement, the follow-up was completed.

### Statistical analyses

The Wilcoxon rank sum test was used to compare the quantitative or ordinal data between groups. Since this study dealt with competing risks (i.e., death, obstruction, and other events), cumulative incidences in each risk were estimated by the Aalen-Johansen approach using the package ‘survival’ and ‘prodlim’ in R according to the reference manual (versions 2.44–1.1. and 2018.04.18, respectively). Open-source software, i.e., R ver. 3.5.2 [27], was used for all statistical analyses. All tests were two-sided, and a *p*-value < 0.05 was considered significant.

## Results

**Fig. 1:**
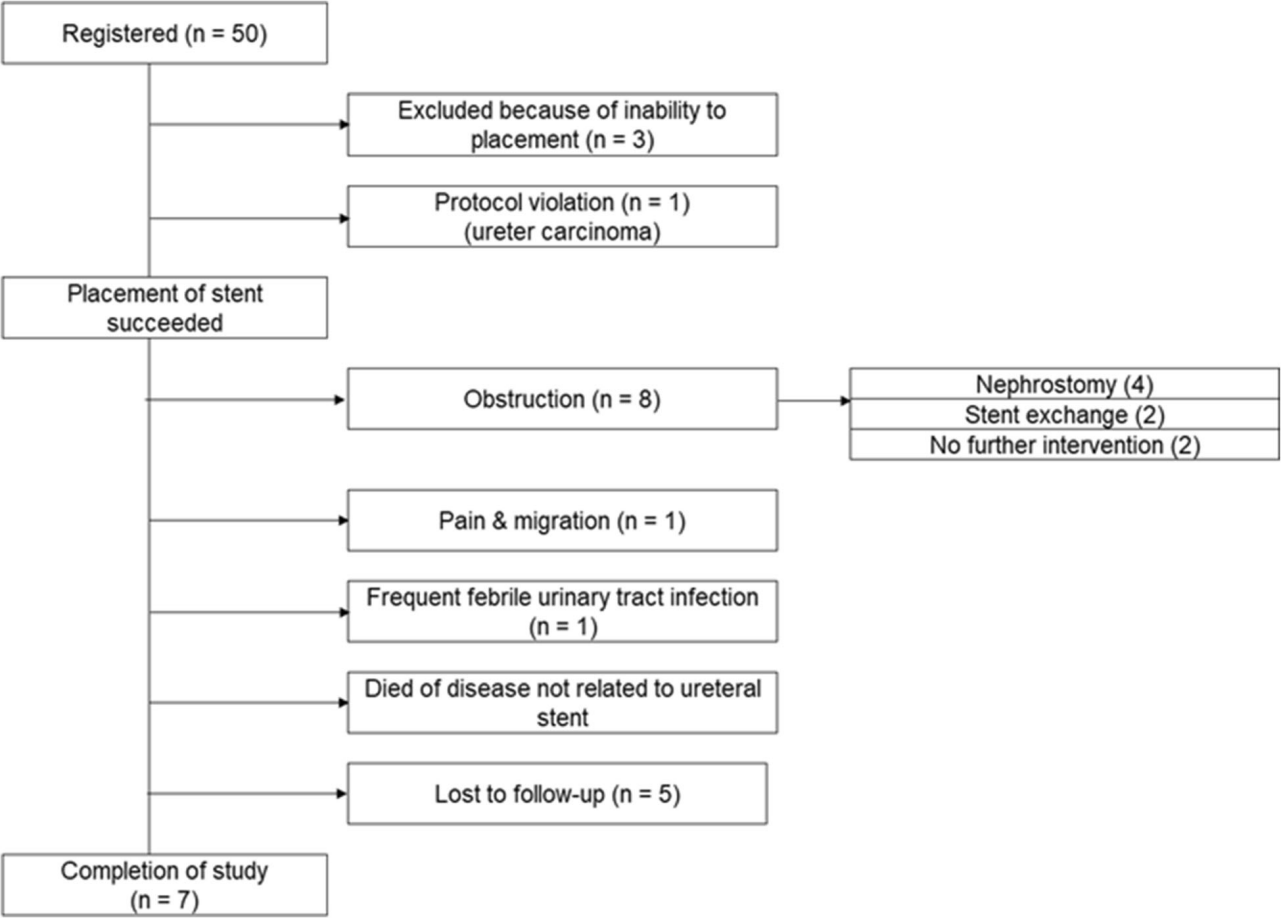
Flow chart with the patient survival/outcomes

A total of 50 patients were enrolled in the study; a Resonance stent was placed in 47 of these patients. Figure 1 is a flow chart with the patient survival/outcomes. In the remaining three patients, a Resonance could not be implanted due to severe ureteral obstruction. With the exception of the three patients with stent insertion failure, there was no problem with the stent insertions. Of the 47 patients, one patient with ureteral cancer was excluded from the analysis because of a protocol violation.

**Fig. 2:**
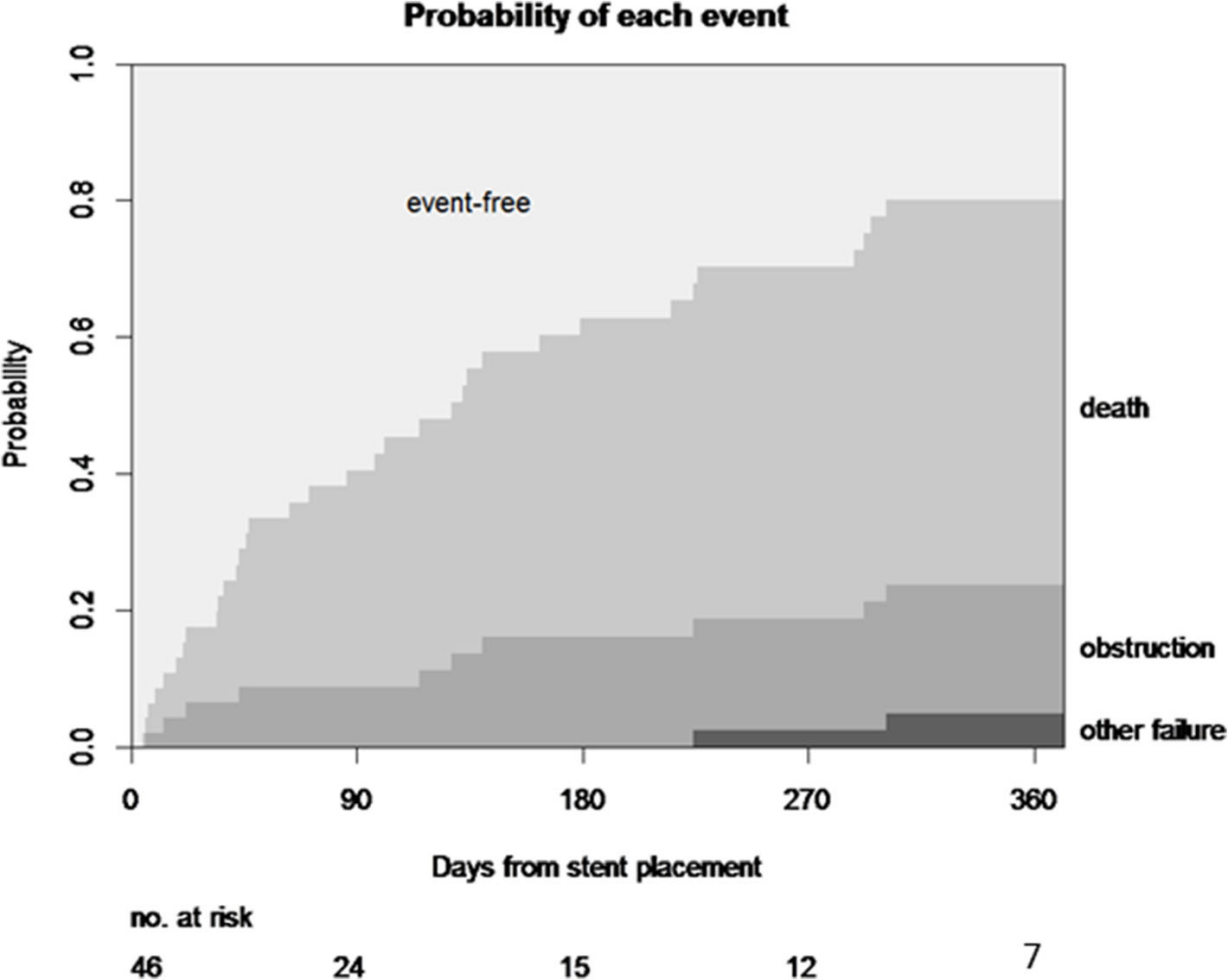
Cumulative incidence curve for each event. The cumulative incidence curves are stacked, and the distance between the two curves at a particular point in time represents the probability of the indicated event

The clinical characteristics of the 46 patients (30 females, 16 males) are summarized in Table 1. The median patient age was 67 years (range 28–85 years). The most common primary malignancy causing the MUO was gastrointestinal cancer (27 patients, 58.7%) followed by gynecological cancer (13 patients, 28.3%). Twenty-six patients had lymph node metastases, ten had peritoneal involvement, and five had bone metastasis. Of the 46 patients, two patients had at least one complication associated with the Resonance (migration, *n* = 1; febrile urinary tract infection, n = 1) and their stents were removed. Twenty-four patients died during the study period. The median overall survival time was 226 days. The median follow-up period for the censored patients was 355 days (range 16–372 days), and just seven patients (six females, one male) were still alive without Resonance failure > 1 year after their stent implantation (Fig. 2).

**Table 1:** Characteristics of the 46 patients with a Resonance stent for MUO

	Total	Female	Male
	(*n* = 46)	(*n* = 30)	(*n* = 16)
Age			
Min.	28	28	43
Median	67	66	68.5
Max.	85	81	85
ECOG performance status			
0	26	16	10
1	14	9	5
2	4	3	1
3	2	2	0
Origin			
Gastrointestinal	27	12	15
Bile/pancreas	6	3	3
Colon	12	7	5
Esophagus	1	0	1
Stomach	8	2	6
Gynecological	13	13	(−)
Ovary	4	4	(−)
Uterine cervix	9	9	(−)
Other	6	5	1
Breast	2	2	0
Malignant Lymphoma	1	1	0
Peritoneum	1	1	0
Prostate	1	(−)	1
Unknown	1	1	0
Metastasis			
No	4	2	2
Yes	42	28	14
Lymph node	26	17	9
Peritoneum	10	7	3
Pelvic organ	3	3	0
Liver	2	1	1
Bone	5	3	2
Lung	7	4	3
Ascites			
No	30	23	7
Yes	16	7	9
Pleural effusion			
No	36	25	11
Yes	10	5	5

Stent obstruction occurred in eight patients: six females and two males (Fig. 1). Although four of these eight patients underwent percutaneous nephrostomy construction and the other two patients were treated by ureteral stent exchange, two patients needed no further intervention because of their short life expectancy. The cumulative stent obstruction incidence at 3, 6, 9, and 12 months after the procedure was 8.9% (95%CI: 0.2–16.8), 16.3% (95%CI: 4.5–26.7), 16.3% (95%CI: 4.5–26.7), and 18.8% (95%CI: 6.1–29.7), respectively (Fig. 2).

**Fig. 3:**
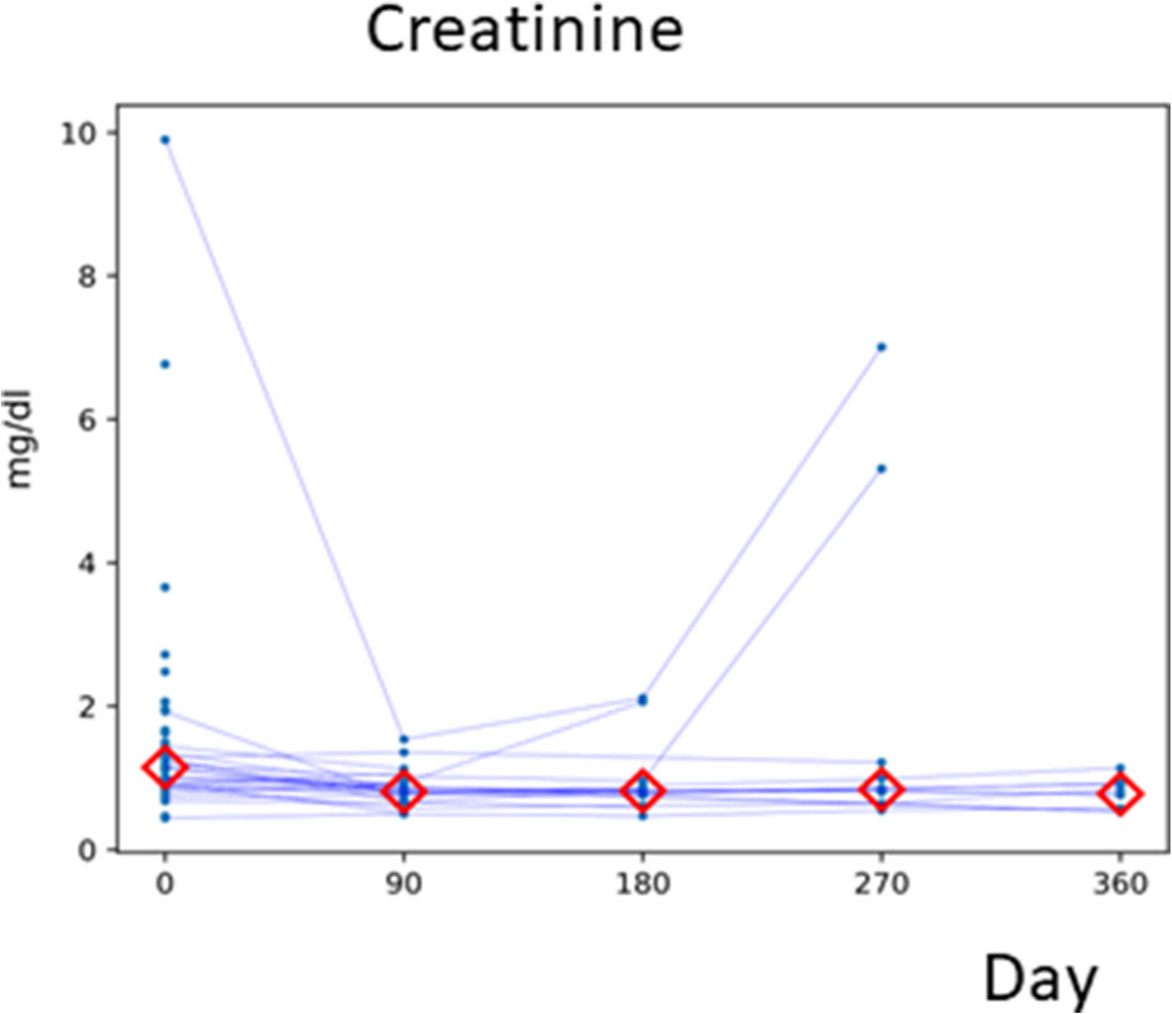
Renal function before (00 M) and after the insertion of a Resonance stent. After insertion, blood samples were collected every 3 months for 12 months. Values ​​from individual patients are shown by lines. The *red diamonds* represent the median at each point in time

The median serum creatinine level before stenting was 1.15 mg/dL (range 0.44–9.90 mg/dL), which decreased to 0.81 mg/dL (range 0.50–1.54 mg/dL) at 3 months post-stenting (*p* = 0.0003) (Fig. 3). In two patients, the creatinine level had risen sharply at 9 months post-stenting, and these two patients died of cancer. The other nine patients had stable creatinine levels up to 12 months (Fig. 3).

**Fig. 4:**
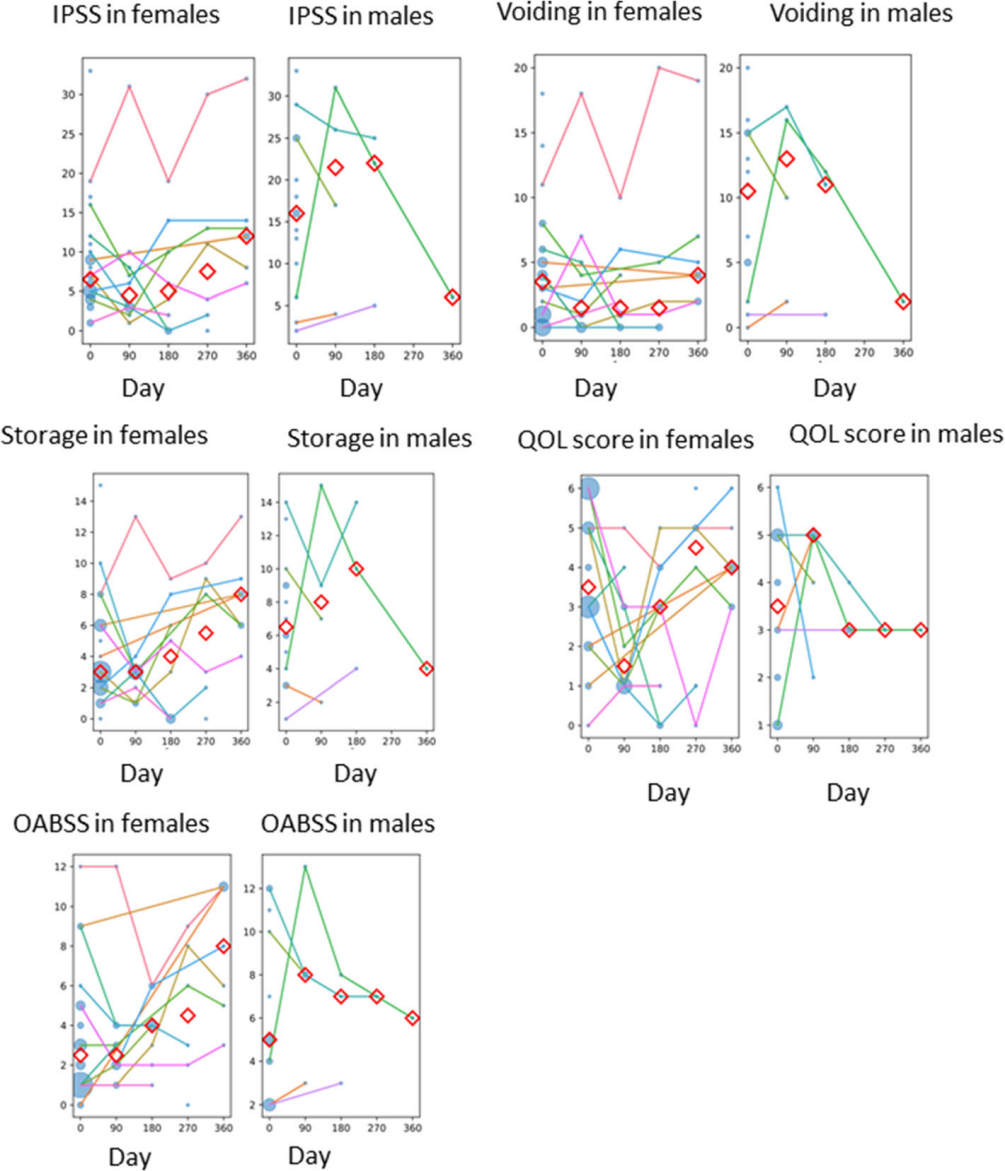
IPSS, storage, and voiding symptom subdomains and the OABSS before and after the insertion of a Resonance stent. After the insertion, symptoms were reported every 3 months for 12 months. Values from individual patients are shown by lines. The *red diamonds* represent the median at each time point (days from intervention). The size of each circle represents the number of patients. Males, *n* = 16; females, *n* = 30

The IPSS scores tended to be lower for the female patients than the males. In particular, the IPSS voiding subscores of the female patients were lower than those of the males, suggesting that the Resonance stent resulted in little discomfort regarding female urination (Fig. 4). Although the female patients’ storage subscores tended to be lower than those of the males, the difference was not significant (Fig. 4). The quality of life (QOL) score was approx. 3 for both the female and male patients (a nonsignificant difference) (Fig. 4). With regard to the OABSS score, although the female patients’ total scores tended to be low, there was not a large difference in total scores between the female and male patients (Fig. 4). The number of male patients in whom the Resonance stent could be left in place until the end of study was only one, and thus the Resonance stent may be painful for male patients.

**Fig. 5:**
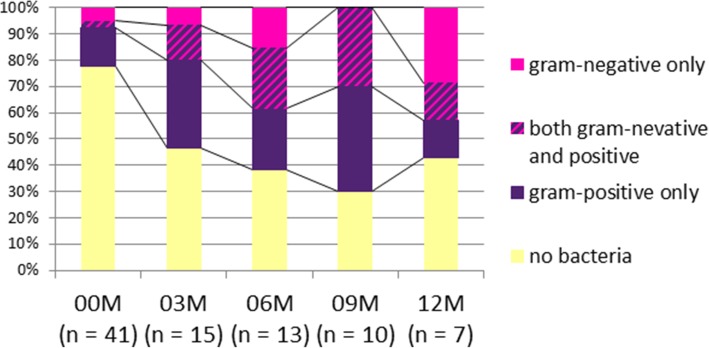
Pathogens isolated from the 46 patients with a Resonance stent

Urine cultures were conducted with 41 samples before stent insertion and 45 samples after stent insertion. The proportion of urine culture-positive patients gradually increased to 9 months after stenting, and approx. 60% of the 46 patients eventually became urine culture-positive (Fig. 5). In 21 patients, one or more urine cultures were conducted after stent placement, but in 25 patients, post-stenting urine culture was not conducted. Among the 21 patients who provided a urine culture after the insertion, 17 (81.0%) experienced at least one positive culture: gram-negative only (*n* = 4), gram-positive only (*n* = 8), and both (*n* = 5). The types of bacteria detected from the urine cultures were more gram-positive than gram-negative strains at all months except 12 months after stent insertion (Fig. 5). Urine cultures were positive in many patients, but despite the positive urine cultures, no fever was observed except in one patient with a febrile urinary tract infection whose Resonance stent was removed.

## Discussion

Since its introduction in 2006, the Cook Resonance® metallic ureteric stent has gained increasing use as an option for relieving ureteric obstruction from a malignant tumor. The management of an MUO is difficult, as urologists must balance renal preservation, patient QOL, and the risk of complications in the setting of a poor prognosis. No consensus has been reached regarding the proper management of MUOs [28, 29]. Earlier studies of the Resonance examined relatively small cohorts, and almost all of the studies were retrospective analyses. We thus examined the treatment outcomes of the Resonance stents for MUO in a prospective study, and we evaluated the relationship between the stent patency rate in the MUO patients and their prognoses. The coiled wire construction of the Resonance metallic stent maintains patency and urinary drainage even under strong exogenous pressure. One of the disadvantages of the use of polymeric stents in Japan is that they must be replaced every 3–6 months, whereas metallic stents can be maintained in place for 12 months before they must be replaced.

Wong et al. reported a minimal benefit regarding overall survival after decompression in patients who had metastases or a previously established diagnosis of MUO [2]. Another study also emphasized that no other factors appeared to play important prognostic roles which patients would benefit from diversion [29]. Asakawa et al. [30] showed that the median survival time of MUO patients was 210 days. Our present analysis revealed the median survival time of 226 days. In clinical settings, the number of survival days for patients with an MUO is expected to be quite low. Considering that the median survival with extrinsic ureteral obstruction as a result of malignancy is < 12 months, stent replacement is probably not necessary during the survival of such patients [3].

Our present findings demonstrated that the treatment success rate for MUO was 77.3% at 6 months and 70.3% at 1 year; these results are similar to those of previous reports [10, 30, 31]. It has also been observed that stent replacement is not necessary in most MUO cases, as we also observed herein. In light of the above-described findings, it is apparent that the frequent replacement of a stent can be avoided, thereby improving patients’ quality of life and reducing medical costs [8, 32].

Medical costs are important to consider when deciding whether to use the Resonance or a conventional polymeric stent. In Japan, stent insertion is permitted by the national health insurance system. The costs of stents are currently as follows: metal ureteral stent, 139,000 yen; polymeric stent, 23,800 yen; and the stent insertion, 34,000 yen. The cost of inserting a polymeric stent twice is 115,600 yen, which is cheaper than a metal ureteral stent, but if a polymeric stent must be used three times, it will be 173,400 yen, which is higher than the cost of inserting the Resonance stent (173,000 yen).

In our study, stent failure occurred in eight (17%) patients. A previous retrospective study [30] reported that 15.4% of their patients experienced stent failure. Moreover, the failure rate of conventional polymeric stents is higher (25–40%) [33]. The Resonance is stiffer than a normal polymeric stent, and it has thus been considered difficult to remove the Resonance. However, in our experience, it was possible to remove the Resonance without adhesion of calculi, and it was also possible to replace the Resonance with a polymeric ureteral stent.

Although the effect of the Resonance stent on patients’ quality of life might vary among individuals, there are no studies using validated questionnaires to assess the stent’s subjective tolerability. In our present cohort, the IPSS voiding subscore was lower in the female patients than the male patients. Indwelling of the stent was considered to have no significant discomfort for urination in the female patients. Although there was not much difference between our female and male patients in the storage subscore or the QOL score, the storage subscore worsened with time in the females. Similarly, the OABSS score did not differ significantly between the female and male patients, but the OABSS scores of the female patients tended to deteriorate over time.

Regarding the patients’ renal function, there was no relationship between the preoperative serum creatinine levels and stent occlusion. Serum creatinine is affected mostly by the patient’s general condition and/or contralateral renal function, and it might not reflect the stent patency rate.

Although bacterial colonization of indwelling ureteral stents is an important issue and colonization rates of 42–90% have been reported [34, 35], in the present study the only significant correlation noted was between urine culture and stenting duration [36]. There is a great discrepancy between urine and catheter cultures [36]. Of the 250 patients in a prior study [37], the risk of bacteriuria and colonization of the ureteral stent tip was significantly associated with the duration of stent retention, patient sex, and the systemic disease (including diabetic nephropathy and chronic kidney disease without dialysis). As mentioned above, in the present study, approx. 81.0% of the patients were positive for urine culture: gram-negative only (*n* = 4), gram-positive only (*n* = 8), and both (*n* = 5). A wide variety of bacteria such as gram-positive bacteria including MRSA and also gram-negative bacteria were detected. However, although the culture results were positive, the patients did not necessarily show signs of infection, and symptoms such as fever related to infection were not observed.

There are several limitations in our study. We could not compare treatment outcomes of the metallic stent and polymeric stents by a randomized control study, as a standard cohort study requires one group of patients with metallic stents and another group with polymeric stents as their initial management. We tried to eliminate potential confounders by conducting a prospective study. This study design also made it possible to determine the patency rate of the Resonance stent in real clinical settings. Second, stent-related symptoms were not evaluated with the use of a Ureteric Stent Symptom Questionnaire (USSQ) [38]. At the time of this study, the Japanese version of the USSQ had not yet been validated. We therefore used the IPSS and OABSS as substitutes. Third, since the length of the stent was determined by the attending physician, symptoms might have been present because the stent was long. Fourth, since there was only one male patient who could be followed up to the end of study, our use of the IPSS score and QOL score cannot provide many conclusions. Fifth, although this was a prospective study, the number of cases was small.

However, compared to other investigations, our study has several advantages. First, the results of this prospective study confirmed that there are many MUO patients with poor prognoses in clinical practice. Second, by using the IPSS, QOL, and OABSS values of MUO patients, we were able to determine the changes in the patients’ quality of life, and we observed that the female patients had less deterioration in the voiding subscore in a real-life setting. Third, although urine cultures from MUO patients are often positive, urine culture positivity did not necessarily represent a serious infection.

In summary, our findings provide a valid argument that a metallic stent is appropriate for MUO patients. It also seems reasonable that a metallic stent should be preferred for female patients. Further research is required to further test metallic stents’ superiority in randomized controlled studies.

## Conclusion

The Resonance stent is effective and safe for relieving MUOs, and 70–80% of our patients with a Resonance stent did not need to have their stent changed at 1 year. In our female patients, the IPSS and OABSS scores were also within self-control during the study period.

## Data Availability

The datasets used and/or analyzed during the current study are available from the corresponding author on reasonable request.

## Abbreviations

IPSS: International Prostate Symptom Score
IPSS-S: IPSS storage subscore
IPSS-V: IPSS voiding subscore
MUO: Malignant ureteral obstruction
OABSS: Overactive Bladder Symptom Score
QOL: Quality of life
T-CReDO: Tsukuba Clinical Research & Development Organization
USSQ: Ureteric Stent Symptom Questionnaire
